# Novel silkworm (*Bombyx mori*) sulfotransferase *sw*SULT ST3 is involved in metabolism of polyphenols from mulberry leaves

**DOI:** 10.1371/journal.pone.0270804

**Published:** 2022-08-04

**Authors:** Kohji Yamamoto, Naotaka Yamada, Satoshi Endo, Katsuhisa Kurogi, Yoichi Sakakibara, Masahito Suiko

**Affiliations:** 1 Department of Bioscience and Biotechnology, Kyushu University Graduate School, Fukuoka, Japan; 2 Laboratory of Biochemistry, Gifu Pharmaceutical University, Gifu, Japan; 3 Department of Biochemistry and Applied Biosciences, Faculty of Agriculture, University of Miyazaki, Miyazaki, Japan; University of Dayton, UNITED STATES

## Abstract

Polyphenols in plants are important for defense responses against microorganisms, insect herbivory, and control of feeding. Owing to their antioxidant, anti-cancer, and anti-inflammatory activities, their importance in human nutrition has been acknowledged. However, metabolism of polyphenols derived from mulberry leaves in silkworms (*Bombyx mori*) remains unclear. Sulfotransferases (SULT) are involved in the metabolism of xenobiotics and endogenous compounds. The purpose of this study is to investigate the metabolic mechanism of polyphenols mediated by *B*. *mori* SULT. Here, we identified a novel SULT in silkworms *(*herein, *sw*SULT ST3). Recombinant *sw*SULT ST3 overexpressed in *Escherichia coli* effectively sulfated polyphenols present in mulberry leaves. *sw*SULT ST3 showed high specific activity toward genistein among the polyphenols. Genistein-7-sulfate was produced by the activity of *sw*SULT ST3. Higher expression of *sw*SULT ST3 mRNA was observed in the midgut and fat body than in the hemocytes, testis, ovary, and silk gland. Polyphenols inhibited the aldo-keto reductase detoxification of reactive aldehydes from mulberry leaves, and the most noticeable inhibition was observed with genistein. Our results suggest that *sw*SULT ST3 plays a role in the detoxification of polyphenols, including genistein, and contributes to the effects of aldo-keto reductase in the midgut of silkworms. This study provides new insight into the functions of SULTs and the molecular mechanism responsible for host plant selection in lepidopteran insects.

## Introduction

Flavonoids and isoflavonoids are a class of polyphenolic secondary metabolites (polyphenols) found in plants that play an important role in defense responses against microorganisms [1], ultraviolet radiation [2], insect herbivory [3], and control of feeding [4]. Owing to their antioxidant, anti-cancer, and anti-inflammatory activities, recognition of their importance in human nutrition has increased in recent years [5]. However, the metabolism of host plant polyphenols in insects remains unclear.

Mulberry tree (*Morus alba*) is a host plant species of the silkworm (*Bombyx mori*), which is a model for lepidopteran species. As a wide variety of polyphenols are found in mulberry trees, these compounds are thought to serve as key chemical cues that enable plants to protect themselves from insects and evaluate their quality as food.

Sulfoconjugation is an important step in xenobiotic detoxification in humans and other organisms. However, to use silkworm as an animal model to study sulfoconjugation reactions, it is essential to identify and characterize various sulfotransferases (SULTs) expressed in silkworm and to analyze the differential sulfating abilities of SULTs for endogenous substrates and xenobiotics. Cytosolic SULTs are members of phase II drug-metabolizing enzymes that transfer a sulfonate group from 3′-phosphoadenosine-5′-phosphosulfate (PAPS) to various substrates (e.g., phenols, enols, alcohols, and amines). In humans, the SULT superfamily comprises more than 60 genes categorized into four SULT families: SULT1, SULT2, SULT4, and SULT6. These SULTs are cytosolic enzymes found in most tissues of our body, including the liver, intestine, brain, adrenal gland, and platelets [6, 7].

Although SULTs have been identified in various organisms, insect SULTs have not been extensively studied, unlike their mammalian counterparts. We have previously studied insecticide metabolism in silkworm and identified a new member of the SULT superfamily, *sw*SULT ST3, as well as three SULTs (bmSULT, *sw*SULT ST1, and *sw*SULT ST2) in *B*. *mori* [8, 9]. The optimal substrates for these enzymes are xanthurenic acid and pentachlorophenol for *sw*SULT ST1 and only xanthurenic acid for *sw*SULT ST2 [8]. However, the optimal substrates for bmSULT remain unknown [9].

This study aimed to elucidate the role of a new SULT identified in silkworms in the metabolism of polyphenols derived from mulberry leaves. We observed that the polyphenols inhibited the activity of *B*. *mori* aldo-keto reductase (AKR2E8), which could play a protective role against reactive aldehydes in the midgut of *B*. *mori* (Yamamoto et al. 2021). Therefore, searching for enzymes that can metabolize polyphenols in silkworms is important. Comprehensive studies on silkworm SULTs may provide insights into the molecular mechanism underlying host plant selection in lepidopteran insects. Therefore, we performed a biochemical characterization of *sw*SULT ST3.

## Materials and methods

### Silkworm and tissue

Silkworms reared on mulberry leaves were obtained from the Institute of Genetic Resources of the Graduate School at Kyushu University (Fukuoka, Japan). Hemocyte, testis, ovary, silk gland, midgut, and fat body required for the experiments were collected on day three from the fifth-instar larvae and stored at −80°C until use. RNeasy Plus Mini Kit (Qiagen, Valencia, CA, USA) was used to extract total RNA from the dissected tissues.

### SULT-encoding cDNA synthesis

Total RNA was used to produce first-strand cDNA using SuperScript II Reverse Transcriptase and an oligo-dT primer. The resulting cDNA was used as a PCR template with the following primers: 5′-ATATATCCATATGCGCACAAAACCGCAATT-3′ (sense for *sw*SULT ST3), and 5′-AAGGATCCTTATTCAAAACTCAGACCGGTG -3′ (antisense for *sw*SULT ST3). The sequences registered in the SilkBase, an expressed sequence tag atabase of silk worm, were referred to for primer design [10]. Sense and antisense primers contained the *Nde*I and *Bam*HI restriction enzyme sites, respectively. PCR conditions consisted of initial denaturation for 2 min at 94°C, followed by 35 cycles of 1 min at 94°C, 1 min at 57°C, and 2 min at 72°C, with a final 10 min extension at 72°C. PCR products were subcloned into the pGEM-T Easy Vector. The GENETYX-MAC software (ver. 14.0.12; https://www.genetyx.co.jp/) was used to obtain the complete gene sequence, determine the amino acid sequence, and calculate the molecular mass and isoelectric point of the protein.

### Quantitative polymerase chain reaction (qPCR)

The qPCR experiments were performed on a *StepOne* Real-Time PCR System (Thermo Fisher Scientific, Waltham, MA, USA). The thermocycling parameters for the qPCR reaction with PowerUp SYBR Green Master Mix consisted of initial denaturation for 2 min at 95°C, followed by 40 cycles of 15 s at 95°C and 60 s at 60°C. Samples were analyzed in triplicates, and the transcript level for the target genes were normalized to their corresponding *rp49* levels. The primers specific for *sw*SULT ST3 and the reference gene (*rp49*) were designed as follows: *sw*SULT ST3 forward, 5ʹ-TGGAGGTACGGGAGAACGAC-3ʹ and reverse, 5ʹ-GCCCGATAAGCCACACCATC-3ʹ; *rp49* forward 5ʹ-CAGGCGGTTCAAGGGTCAATAC-3ʹ, and reverse 5ʹ-TGCTGGGCTCTTTCCACGA-3ʹ.

### Protein preparation

The pGEM-T Easy Vector carrying *SULT* was cut with *Nde*I and *Bam*HI. The *sw*SULT ST3 insert was ligated into the pMAL-c5X expression vector and transformed into BL21 competent *E*. *coli* cells. Transformed BL21 cells were cultured at 37°C in Luria–Bertani (LB) medium (BD Biosciences, Franklin Lakes, NJ, USA) supplemented with 100 μg/mL ampicillin until OD_600_ reached 0.5. After treatment with isopropyl β-D-1-thiogalactopyranoside (IPTG) at a final concentration of 0.5 mM, the cells were incubated at 28°C overnight to induce the production of the recombinant maltose-binding protein (MBP)-SULT fusion protein. Cells were collected by centrifugation and disrupted by sonication using an ultrasonic disruptor (TOMY, Tokyo, Japan). The prepared supernatants were applied to amylose resin (New England Biolabs, Boston, MS, USA). After washing with the column buffer (20 mM tris-HCl at pH 8.0, 0.2 M NaCl, and 1 mM EDTA), the MBP-*sw*SULT ST3 fusion protein was eluted using a stepwise maltose gradient (1, 2.5, 5, and 10 mM) in the column buffer. The eluates were desalted using a centrifugal filter (Merck Millipore; *Burlington*, MA, USA) and applied to a Superdex 200 column (GE Healthcare, Little Chalfont, UK). The purity of the eluates was examined using sodium dodecyl sulfate polyacrylamide gel electrophoresis (SDS-PAGE), followed by Coomassie Brilliant Blue R250 staining. Protein concentration was determined using the Bradford method [11].

### Measurement of SULT activity

Thin-layer chromatography (TLC) was performed in the presence of PAP[^35^S] as the sulfate donor in the presence of several substrates [12]. The reaction mixture containing each substrate (25 μM), *sw*SULT ST3 (1.0–2.0 μg/20 μL), and HEPES buffer (pH 7.0) was incubated at 37°C for 10 min. The reaction was stopped by heating. Then, 1 μL of the reaction mixture was applied to a cellulose or silica gel TLC plate using *n*-butanol/ isopropanol/formic acid/ water (3:1:1:1 by volume) as the solvent system to separate the sulfated products. We detected the spot positions corresponding to the sulfated products on the TLC using a Fluoro Image Analyzer, Typhoon FLA 9500 (Cytiva, Tokyo, Japan). The specific activity of *sw*SULT ST3 towards various substrates was measured using a phosphatase-coupled assay with a Universal Sulfotransferase Activity Kit (R&D Systems, Minneapolis, MN, USA) in the presence of non-labelled PAPS. The assay was composed of two steps: (1) conversion of PAPS to PAP by *sw*SULT ST3, and (2) release of inorganic phosphate by a coupling phosphatase. After incubation at 37°C for 1 h, the change in absorbance was detected at 620 nm.

### AKR2E8 preparation

Recombinant AKR2E8 was prepared as previously described [13]. Briefly, a pET-15b expression vector (Merck Millipore, Burlington, MA, USA) carrying the *akr2e8* insert was transformed into competent *E*. *coli* BL21 (DE3) pLysS cells (Toyobo, Osaka, Japan). Transformed cells were incubated at 37°C in LB medium containing 100 μg/mL ampicillin. The recombinant protein was induced by the addition of 1 mM IPTG (Wako, Osaka, Japan). After culturing for 3 h at 28°C, the cells were centrifuged at 5,000 ×*g* at 4°C and mixed with 20 mM Tris-HCl containing 0.5 M NaCl (pH 8.0), 4 mg/mL lysozyme (Wako, Osaka, Japan), and 1 mM phenylmethanesulfonyl fluoride, after which they were sonicated three times for 20 s each under specific conditions (output control 4 and duty cycle 50%) on ice using an ultrasonic disruptor (TOMY, Tokyo, Japan).

Cell lysates were isolated by centrifugation at 10,000 ×*g* for 15 min, and the supernatant was mixed with nickel-affinity resin (GE Healthcare) and incubated for 30 min at 4°C with shaking. The suspension was loaded onto a polypropylene column (Bio-Rad, Hercules, CA, USA), washed with 20 mM Tris-HCl (pH 8.0) containing 0.5 M NaCl, and the bound samples were eluted using 0.5 M imidazole in the same buffer. Eluates were desalted by a centrifugal filter (Merck Millipore) and applied to a Superdex 200 column (GE Healthcare).

The IC_50_ values for AKR2E8 toward polyphenols were determined in a reaction mixture (2.0 mL), including 0.1 M potassium phosphate (pH 7.4), 0.1 mM NADPH, 25 μM benzaldehyde, and the enzyme. AKR2E8 was prepared as previously described (Yamamoto et al. 2021). AKR2E8 activity in the presence of each polyphenol was assayed by NADPH-linked benzaldehyde reduction in the presence of 0.1 mM NADPH. The IC_50_ values are expressed as the means ± SD of at least three determinations.

### Analysis of the metabolites of swSULT ST3 reaction

The ability of *sw*SULT ST3 to metabolize genistein was determined using liquid-chromatography-tandem mass spectrometer (LC-MS/MS). Briefly, the reaction mixture (100 μ L) containing 25 μM genistein, 0.5 μM PAPS, and 2 μg *sw*SULT ST3 in 10 mM HEPS buffer (pH 7.0) was incubated at 30°C for 30 min. After incubation, the mixture was boiled for 3 min and diluted with 100 μL of methanol for LC-MS/MS analysis. The metabolites produced by the activity of *sw*SULT ST3 was identified using an LCMS-IT-TOF mass spectrometer (Shimadzu Inc., Kyoto, Japan). The reacted sample (5 μL) described above was subjected to reverse-phase HPLC on a CAPCELL PAK C18 column (150 × 3.0 mm i.d.; Shiseido Fine Chemicals, Tokyo, Japan) at 40°C, with 0.1% (v/v) formic acid/H_2_O containing 5 mM ammonium formate as solvent A and 0.1% (v/v) formic acid/methanol as solvent B at a flow rate of 0.20 mL/min. The metabolite was eluted on a linear solvent gradient program using the following steps: 30% solvent B for 1 min 30–100% solvent B for 7 min, and 100% solvent B for 13 min to analyze the reaction products. The electrospray ionisation (ESI negative) source was used in the MRM mode. The MS was operated using the following parameters: probe voltage, 1.70 kV; collision-induced dissociation temperature, 200°C; block heater temperature, 200°C; nebulizer gas (nitrogen) flow, 1.5 L/min; ion accumulation time, 50 ms; tolerance, 0.05 m/z; MS range of m/z, 100–1000; and MS/MS range of m/z, 100–1000. The IT-TOF mass spectrometer was operated in the data-dependent acquisition mode using LCMS-solution version 3.50 software (Shimadzu).

### Statistical analysis

One-way ANOVA was used to determine the differences between each group. A *p* value of < 0.05 was considered statistically significant.

## Results

### Cloning and sequencing of swSULT ST3

cDNA encoding *sw*SULT ST3 from the larval fat body of *B*. *mori* was generated using reverse-transcription PCR. The nucleotide sequence was determined and deposited in GenBank (accession number: LC595230). The open reading frame of the *sw*SULT ST3 gene encodes 331 amino acids (S1 Fig); the calculated molecular weight of the protein was 38,563, and its isoelectric point was 6.02 (Table 1). The *sw*SULT ST3 shares 30%, 28%, 28%, 25%, 27%, and 25% similarity with human ST4A1, human ST6B1, swSULT ST1, swSULT ST2, bmST1, and bmSULT, respectively (Table 1, S1 Fig). The amino acid residues for the 5′-phosphosulfate binding loop (5′-PSB loop), the 3′-phosphate binding motif (3′-PB motif), and the P-loop were conserved in the SULT sequences and are underlined (S1 Fig). Based on the SULT phylogenetic tree, *sw*SULT ST3 is closely related to mammalian SULT4As (S1 Fig).

**Table 1:** Properties of *Bombyx mori* sulfotransferase (SULT) as determined by the present and previous studies [8, 9].

Properties	swSULT ST3	swSULT ST1	swSULT ST2	bmSULT
Sequence homology (%)	100	28	28	27
Molecular weight	38,563	38,172	33,887	35,789
Isoelectric point	6.0	6.3	5.9	8.4
Tissue distribution	Midgut, fat body	Midgut, ovary	Ovary, testis	Midgut
Substrate specificity	Genistein, quercetin, xanthurenic acid	Xanthurenic acid, pentachlorophenol	Xanthurenic acid	Unknown

### Tissue distribution of swSULT ST3 mRNA

**Fig 1:**
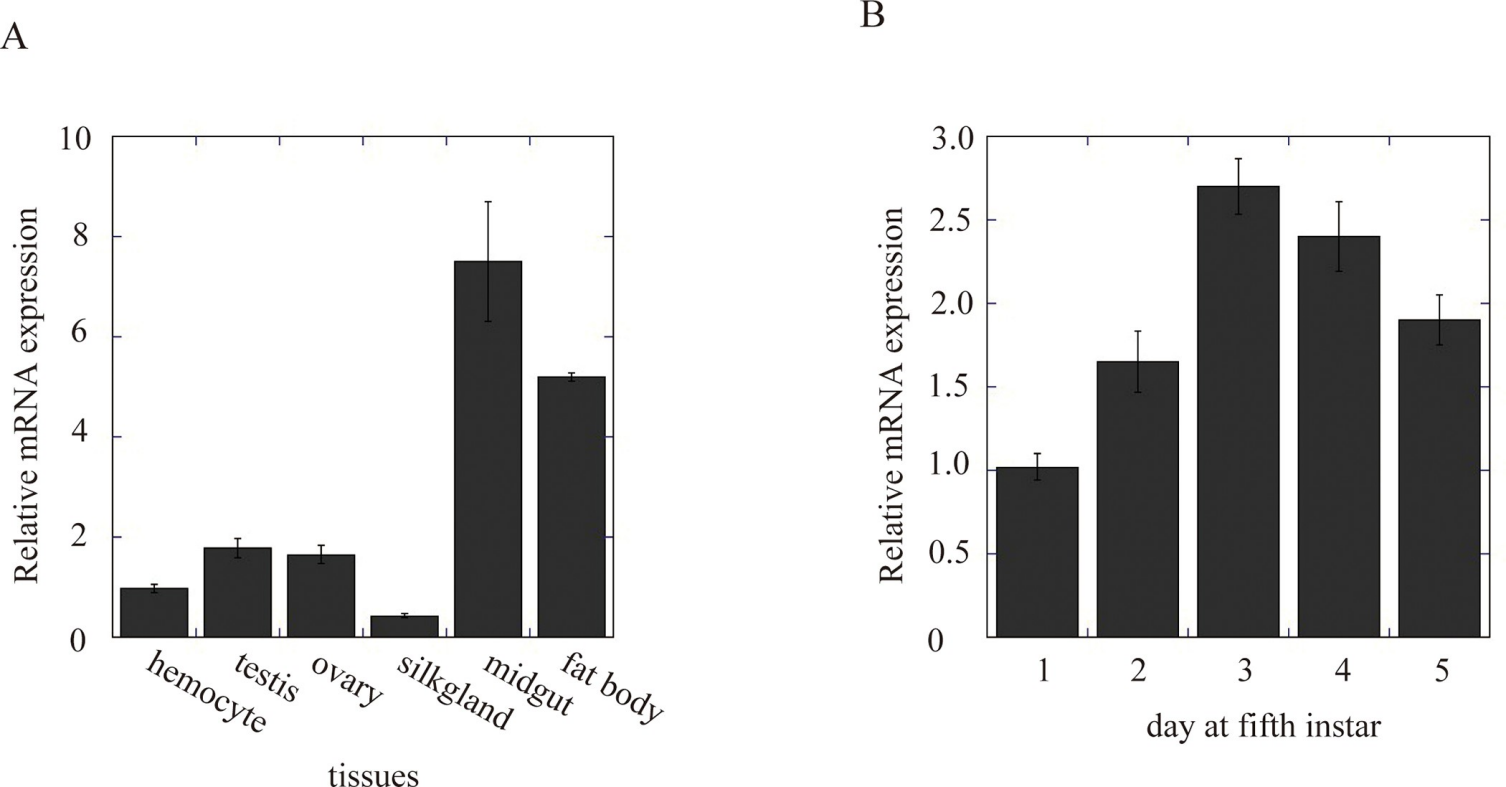
*SULT* mRNA relative expression. (A) *sw*SULT ST3 mRNA was detected using qPCR in different tissues of *Bombyx mori*. The corresponding values are relative to that of reference gene *rp49*. (B) Time-dependent expression of *sw*SULT ST3 mRNA. Error bars indicate the standard deviation of three separate experiments.

The relative *sw*SULT ST3 mRNA expression in various tissues (Fig 1) was investigated using qPCR. Although the *sw*SULT ST3 transcript was present in all silkworm tissues tested, it was most abundant in the midgut and fat body (Fig 1A, Table 1). The highest amount of *sw*SULT ST3 transcript was observed on day 3 at the fifth larval instar (Fig 1B).

### swSULT ST3 characteristics

**Fig 2:**
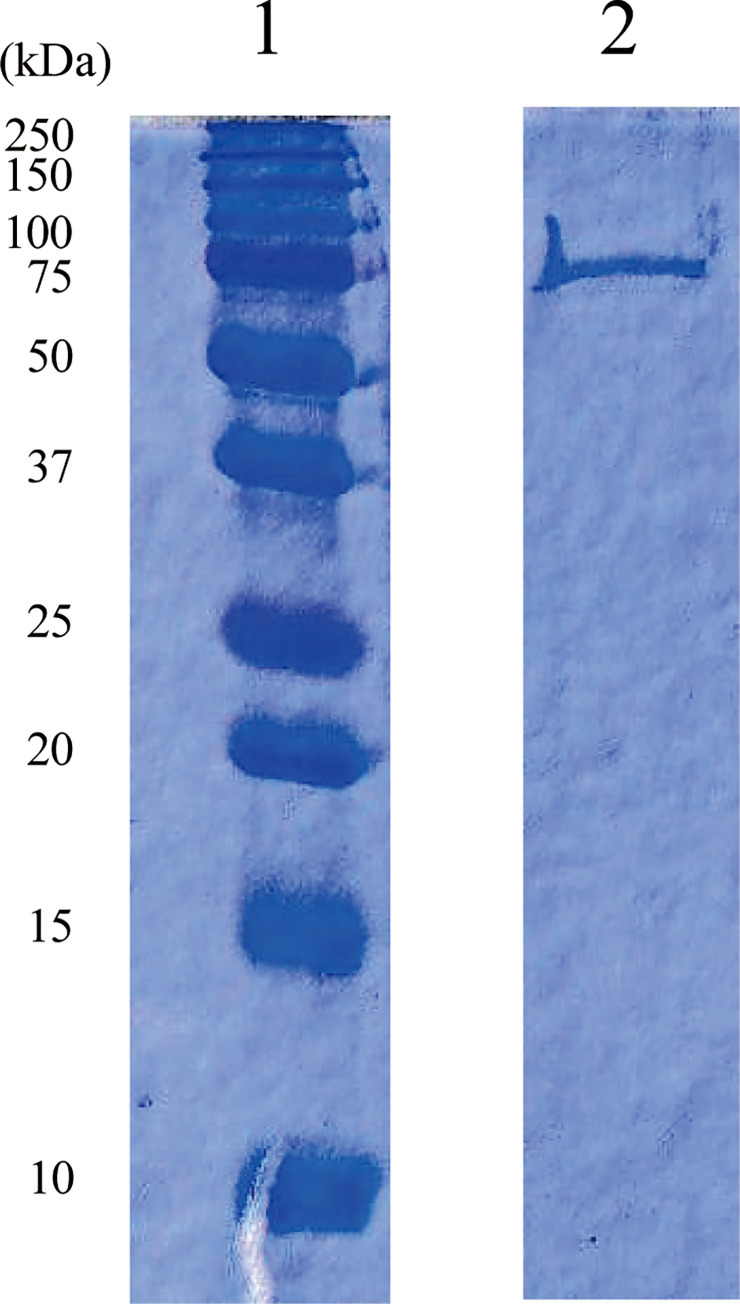
SDS-PAGE analysis of purified *B*. *mori sw*SULT ST3. Proteins were run on a 15% polyacrylamide gel and stained with Coomassie Blue. Lanes 1 and 2 correspond to molecular size markers and *sw*SULT ST3 after Superdex 200 column, respectively. Molecular sizes are shown on the right.

**Fig 3:**
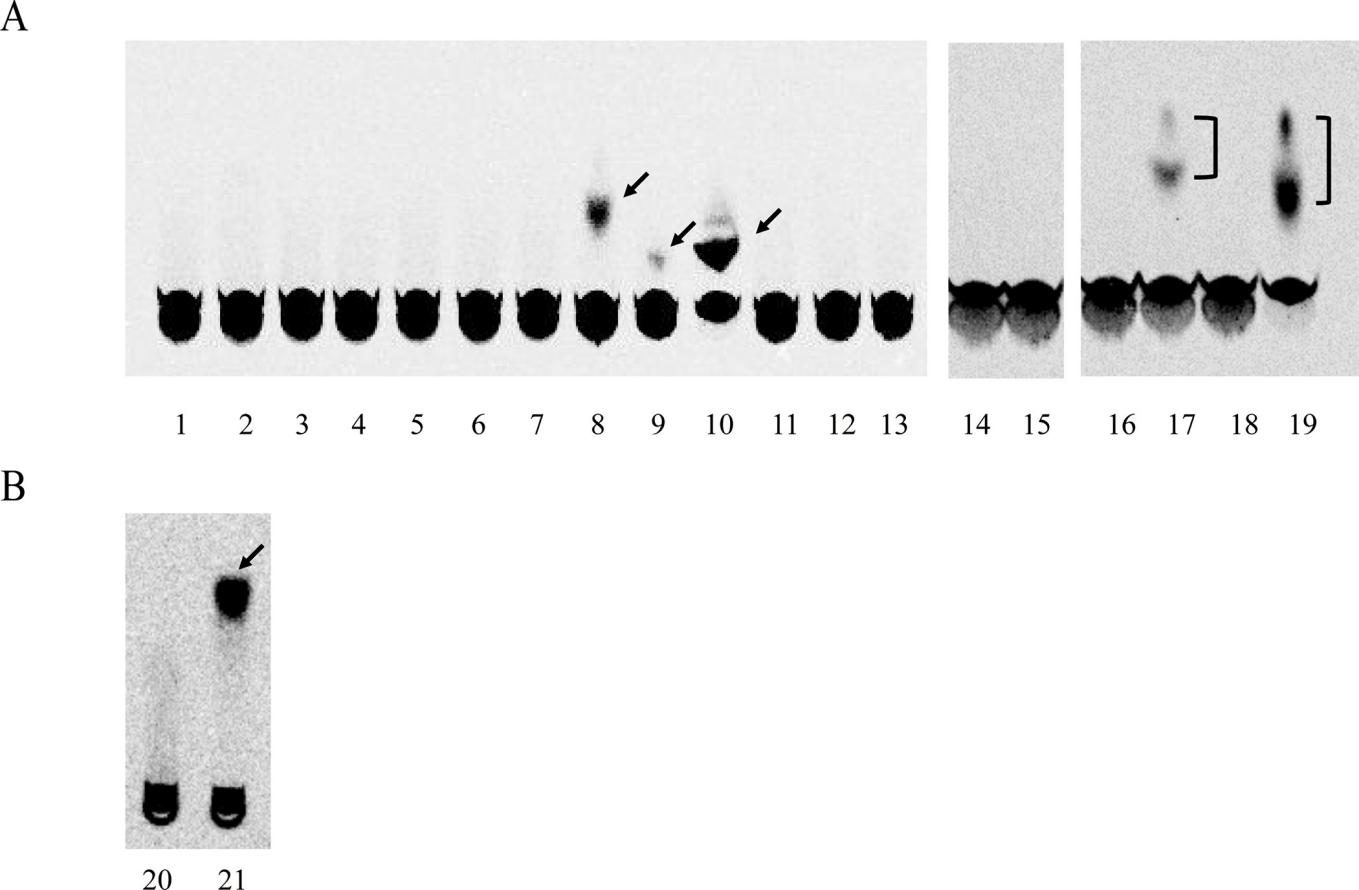
Thin-layer chromatography (TLC) of [^35^S] sulfated derivatives produced by *sw*SULT ST3 with various substrates. Representative autoradiographs derived from TLC plates after elution are shown here. Lane numbers for (A) and (B) indicate 1) DMSO, 2) *p*-nitrophenol, 3) 1-naphthol, 4) naphthylamine, 5) dopamine, 6) serotonin, 7) triiodothyronine, 8) daidzein, 9) resveratrol, 10) xanthurenic acid, 11) 17β-estradiol, 12) dehydroepiandrosterone, 13) corticosterone, 14) chlorogenic acid, 15) caffeic acid, 16) rutin, 17) genistein, 18) catechin, 19) apigenin, 20) DMSO, and 21) quercetin. Cellulose (A) and silica gel (B) TLC plates were used. The arrows and square brackets indicate the sulfated products of each substrate.

To better understand the enzymatic characteristics of *sw*SULT ST3, we purified the recombinant forms (Fig 2). Analysis of *sw*SULT ST3 using SDS-PAGE revealed a single protein band (Fig 2). The activity of *sw*SULT ST3 was screened by performing sulfation assays with the following 19 substrates: *p*-nitrophenol, 1-naphthol, naphthylamine, dopamine, serotonin, triiodothyronine, 17β-estradiol, dehydroepiandrosterone, corticosterone, xanthurenic acid, daidzein, resveratrol, chlorogenic acid, caffeic acid, quercetin, rutin, genistein, catechin, and apigenin (Fig 3A and 3B). Of these, *sw*SULT ST3 effectively sulfated daidzein, resveratrol, xanthurenic acid, quercetin, genistein and apigenin, whereas no activity was detected with the other 13 substrates (Fig 3A and 3B). Determination of the specific activity of *sw*SULT ST3 (Table 2) revealed that the enzyme showed higher activity for genistein, xanthurenic acid, quercetin, and apigenin, with an approximately 30- to 41-fold and 10- to 14-fold higher activity than that of resveratrol and daidzein, respectively.

**Table 2:** Substrate specificities of *sw*SULT ST3.

Substrate	Substrate specificity (pmol/min/mg)
Daidzein	11 ± 2.1
Resveratrol	3.7 ± 1.8
Xanthurenic acid	14 × 10 ± 25
Quercetin	14 × 10 ± 26
Genistein	15 × 10 ± 12
Apigenin	11 × 10 ± 7.5

### Inhibition of AKR2E8 by polyphenols

The effect of polyphenols on AKR2E8 activity was examined (Table 3). Among the polyphenols, the most prominent IC_50_ value was obtained in the presence of genistein.

**Table 3:** Inhibitory effects of polyphenols on aldo-keto reductase (AKR) 2E8 activity.

Inhibitors	IC_50_ (μM)
Genistein	0.018 ± 0.0014
Daidzein	0.034 ± 0.0027
Quercetin	0.065 ± 0.0018
Apigenin	0.17 ± 0.010
Kaempferol	0.46 ± 0.12
Resveratrol	0.55 ± 0.023

### Identification of metabolites produced by the activity of swSULT ST3

**Fig 4:**
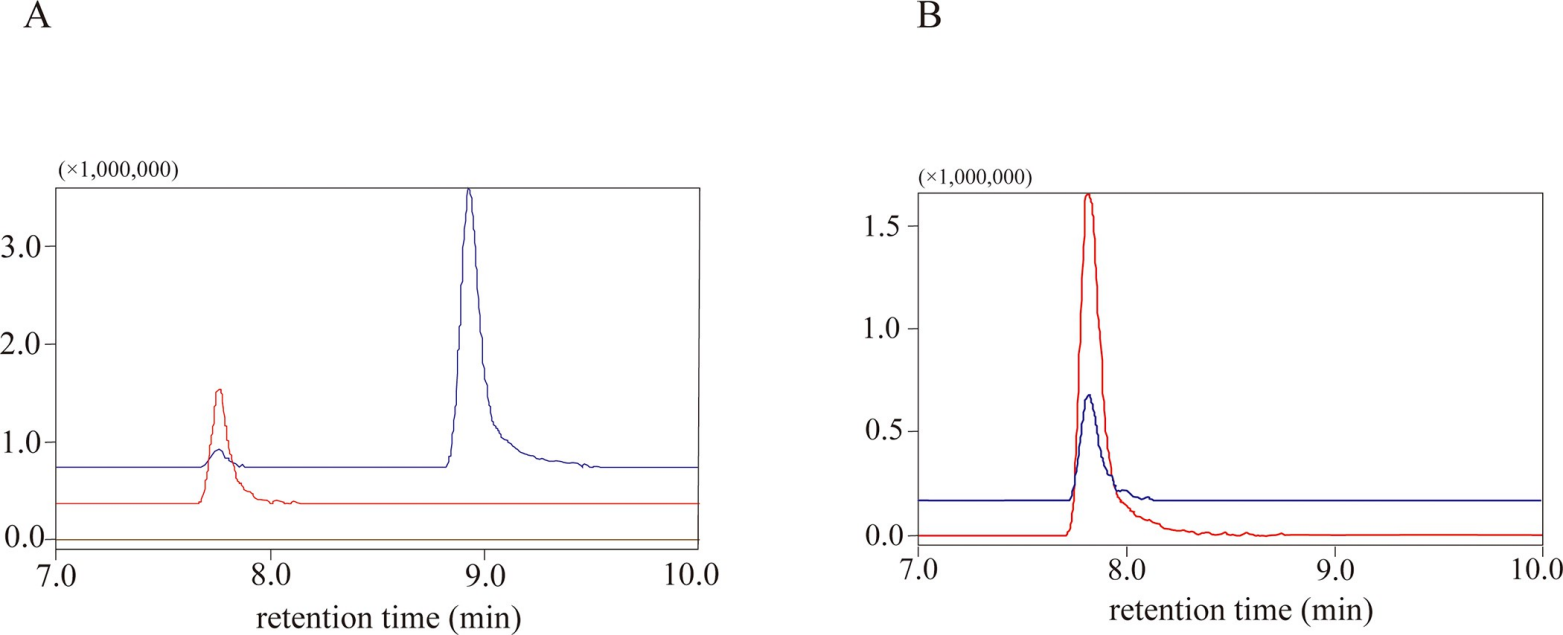
Analysis of reaction products of *sw*SULT ST3 using liquid-chromatography-tandem mass spectrometry (LC-MS/MS). Mass chromatograms are shown on selective mass traces at m/z 269.0450 (blue), m/z 349.0018 (magenta) and m/z 428.9256 (brown). The reaction mixture after incubation of genistein with *sw*SULT ST3 and PAPS (A) and genistein-7-sulfate standard (B) were loaded onto LC-MS/MS. Chromatographic conditions are described in the Methods section. Genistein, the reaction product, and genistein-7-sulfate are indicated by an arrow.

**Fig 5:**
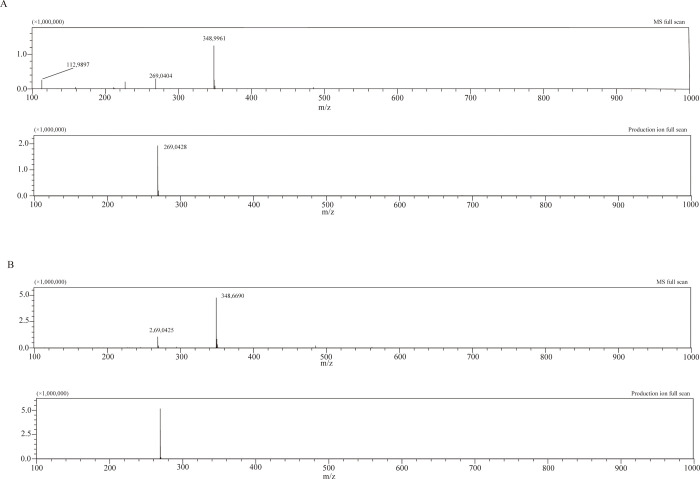
Negative ion mass-spectrometry (MS) spectra and tandem MS product ions spectra of genistein metabolite and genistein-7-sulfate of *sw*SULT ST3. Analysis of the genistein metabolite (A) and genistein-7-sulfate (B) was carried out. Each panel includes the result of MS full scan and production ion scan at m/z 349.0018.

We examined the metabolites produced by *sw*SULT ST3 activity using LC-MS/MS under three conditions at m/z 269.0450, m/z 349.0018 and m/z 428.9256. Two peaks at 7.3 min and 8.9 min in detection at m/z 269.0450 were observed after the analysis of the reaction mixture (Fig 4). The peak at m/z 349.0018 had a retention time of 7.3 min and matched with that of genistein-7-sulfate (Fig 4). Similarly, the retention time of the peak at 8.9 min was the same as that of genistein. No peak was detected at m/z 428.9256. Negative MS analysis of the peak at 7.3 min showed the precursor ion m/z 348.9961[M-H]^-^ (calculated molecular weight of genistein-sulfate: 350.0096) (Fig 5A). Furthermore, MS analysis of the fragments of the peak at 7.3 min revealed ions at m/z 269.0428 [M-H]^-^ (Fig 5A). Similarly, the precursor ion and the fragmentation pattern for genistein-7-sulfate revealed a precursor ion (Fig 5B) and a fragmented product ion at m/z 348.9960 [M-H]^-^ and 269.0422 [M-H]^-^ (Fig 5B), respectively.

## Discussion

**Fig 6:**
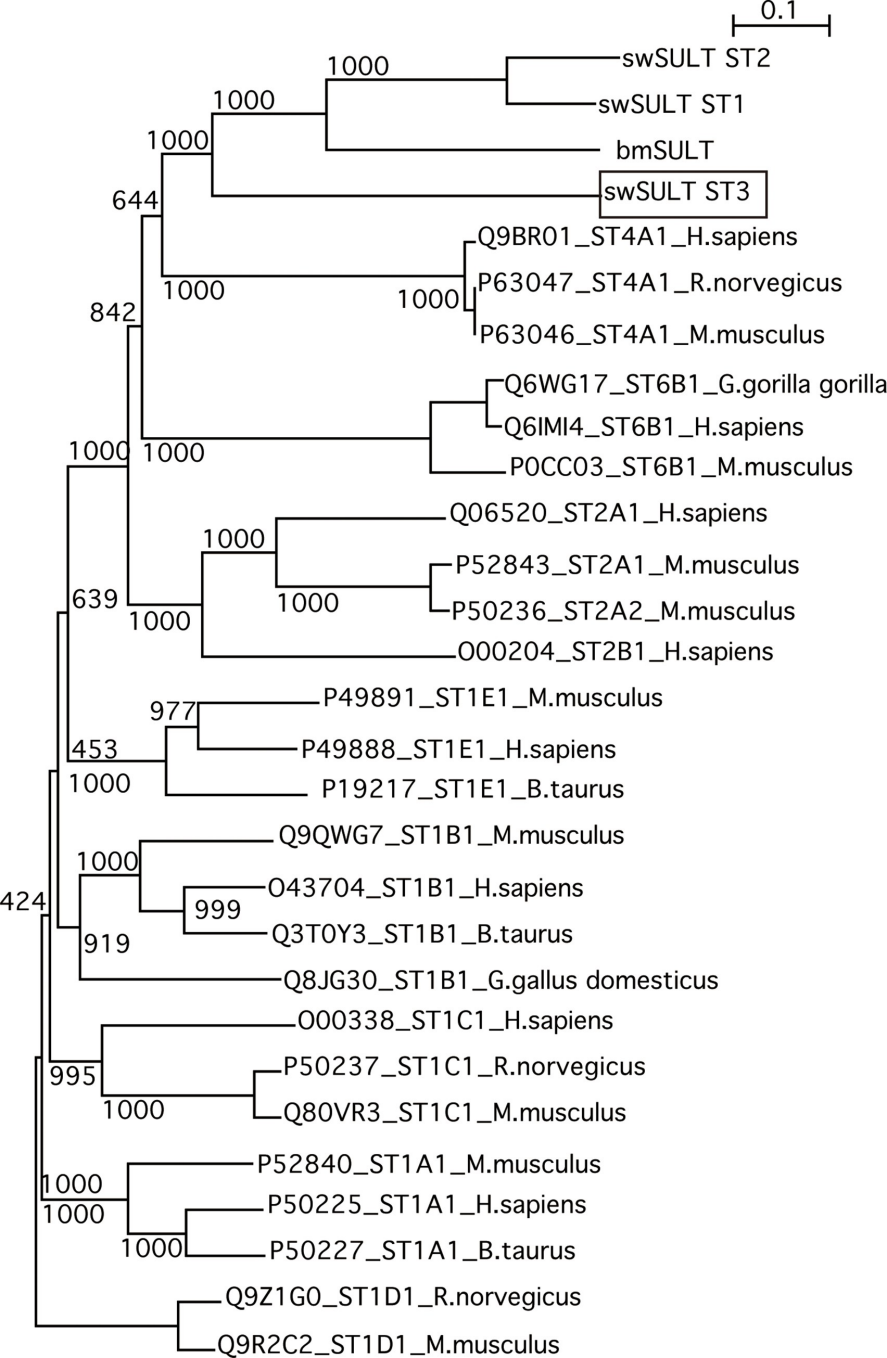
Phylogenetic analysis of sulfotransferase (SULT) amino acid sequences. Primary amino acid sequence comparisons were performed by the neighbor-joining method using various SULT sequences retrieved from the Swiss-Prot database (http://web.expasy.org/docs/swiss-prot_guideline.html). The bootstrap value for each node is shown.

SULTs are involved in the sulfate conjugation of endogenous compounds and xenobiotics [14]. The draft genome sequence of the silkworm indicates that there are 27 potential SULTs. Among them, we identified three SULTs of *B*. *mori* [8, 9]. Compared with SULTs from other species, what is known about the functions of SULTs in *B*. *mori* is limited. In this study, we reported a novel SULT of *B*. *mori*, which we have designated as *sw*SULT ST3. Although the amino acid sequence of swSULT3 showed 27–28% homologies to those of other SULTs of *B*. *mori*, amino acid residues required SULT activity are conserved. For example, specific amino acid residues (5′-PSB loop, 3′-PB motif, and P-loop) interact with PAPS molecules, leading to catalysis by *sw*SULT ST3 (S1 Fig). Although phylogenetic tree analysis revealed that *sw*SULT ST3 was the most similar to SULT4As among the known enzymes (Fig 6), SULT4As displayed lower activity towards known substrates [15]. Thus, we generated a recombinant protein using bacterial expression to investigate the function of *sw*SULT ST3. After the MBP was removed, precipitation of the recombinant protein was observed. Since MBP alone did not exhibit sulfation activity against xanthurenic acid [8] and genistein, the fusion protein was used in the further experiments. The results indicated that *sw*SULT ST3 could catalyze the sulfation of polyphenols listed in Table 2. In contrast, neither *sw*SULT ST1 nor *sw*SULT ST2 possessed this function. No activity was detected for the other tested substrates, including *p*-nitrophenol, 1-naphthol, naphthylamine, dopamine, serotonin, triiodothyronine, 17β-estradiol, dehydroepiandrosterone, and corticosterone. Variations in the catalytic amino acid residues between *sw*SULT ST3 and other SULTs were observed which could play a role in substrate specificity by favoring certain conformational changes within each enzyme.

swSULT ST3 mRNA is highly expressed in midgut, like swSULT ST1 and bmSULT (Table 1). The silkworm midgut is the central tissue for nutrient absorption; furthermore, it serves as a barrier to prevent exogenous substances from entering the cells. Mulberry leaves contain polyphenols such as quercetin, apigenin, resveratrol, daidzein, and genistein [16–20], all of which have biological activity. For example, quercetin is used as an antioxidant to reduce the immunosuppressive effects of hyperglycemic sera in patients with type 2 diabetes [21]. Similarly, apigenin has antioxidant, anti-inflammatory, and antitumor functions [22]. In addition, resveratrol exhibits antitumor and phytoestrogenic activities [23]. Genistein is structurally similar to estrogen, thereby, functionally sharing estrogen action [24]. Thus, several reports on the effects of plant polyphenols in mammalians are being reported. In contrast, little is known about their effect in insects. In this study, the highest expression of *sw*SULT ST3 was observed on day three of the fifth larval instar in silkworms; furthermore, we believe it is likely to be involved in metabolizing polyphenols in the midgut tissues.

The AKR superfamily comprises approximately 190 proteins that catalyze nicotinamide adenine dinucleotide phosphate (NADPH)-dependent reactions and participate in various physiological processes [25]. We have identified some AKRs of *B*. *mori* [13, 26–28]. One of them, AKR2E8, is present abundantly in *B*. *mori* midgut [13] and mulberry polyphenols inhibit its activity (Table 3). Similar observations were made in humans. Polyphenols from *Rhus verniciflua* show inhibitory activity of AKR 1B10 from humans [29]. Plant polyphenols inhibit human cytochrome P450, another detoxifying enzyme [30]. AKR2E8 reduced benzaldehyde, hexanal, heptanal, nonanal, trans-2-nonenal, and citral present in mulberry leaves. In addition, we observed the highest expression of AKR2E8 mRNA in the midgut on day three of the fifth larval instar (Fig 1). Genistein is a specific inhibitor of tyrosine-kinase [31]. In *B*. *mori*, effect of genistein on the activity of prothoracicotropic hormone in prothoracic glands was reported [32]. Together with inhibition of AKR2E8 by genistein, it is crucial to control the mode of action of genistein in *B*. *mori*. Since the highest expression of *sw*SULT ST3 was also observed on day three of the fifth larval instar, AKR2E8 apparently works in coordination with *sw*SULT ST3 in the detoxification of reactive aldehydes in the midgut of silkworm.

LC-MS analysis of the reaction products of *sw*SULT ST3 revealed that the retention time of the peak at 7.3 min was identical to that of genistein-7-sulfate. MS full-scan (Fig 5A and 5B) showed that the highest relative abundance for both the metabolite corresponding to the peak at 7.3 min (m/z 348.9961) and genistein-7-sulfate (m/z 348.9960) corresponded to the theoretical monoisotopic mass of mono-sulfated genistein. As shown in the production ion full scan chromatogram (Fig 5A and 5B). Further, selected ion monitoring of each parent ion and the subsequent high-energy collision induced fragmentation of the parent ions generated fragments of m/z 269.04 which correspond to genistein lacking one SO_3_^-^ group (m/z 79.96). This suggests that the metabolite was mono-sulfated genistein. Similar observation was reported for human SULT1C4 which catalyzed the production of genistein 7-sulfate [33].

Similar to swSULT ST1 and ST2 (Table 1), swSULT ST3 can catalyze xanthurenic acid. Xanthurenic acid is a metabolic intermediate in the kynurenine pathway, by which nicotinamide adenine dinucleotide is produced. This pathway includes tryptophan, kynurenine, and 3-hydoroxykynurenine as metabolic intermediates. Xanthurenic acid has been reported to occur in silkworms [34]. Furthermore, the production of ecdysone is reduced by xanthurenic acid in crayfish and silkworms [35]. Previously, we have reported that both *sw*SULT ST1 and *sw*SULT ST2 are able to sulfate xanthurenic acid [8]. Therefore, we propose that similar to the activities of these two enzymes, *sw*SULT ST3 might regulate the amount of xanthurenic acid in silkworms.

We observed differences in substrate specificity among *B*. *mori* SULTs (Table 1). Based on the amino-acid alignment (S1 Fig), we found that some replacements in amino acid residues for enzyme catalysis. For example, Gly48 in swSULT ST3 was replaced with Arg in swSULT ST1 and ST2. Ile51 in swSULT ST3 was substituted with Thr swSULT ST1 and ST2. It may be necessary for other part in amino acid of swSULT ST3 to the catalytic function. We are currently preparing swSULT ST3 crystals for X-ray crystallography. Studies on structure–function analysis and substrate-binding specificity would provide more knowledge for understanding the differences in substrate specificity.

Generally, sulfoconjugation increases the polarity and water-solubility of a molecule, thereby facilitating detoxification and biliary or urinary excretion of numerous compounds [36, 37]. The tissue-specific expression of *sw*SULT ST3 in the silkworm described herein and its activity towards different substrates indicate that *sw*SULT ST3 likely plays a role in the metabolism of polyphenols in the silkworm midgut and in the detoxification of xanthurenic acid in the silkworm fat body. Genome-editing will further allow the determination of the physiological roles of *sw*SULT ST3 in *B*. *mori*.

## Supporting information

S1 Fig *Bombyx mori* sulfotransferase (SULT) sequence alignment. SULT sequences from various species were retrieved from the Swiss-Prot database (http://web.expasy.org/docs/swiss-prot_guideline.html). Boxes enclose the amino acid residues that interact with PAPS molecules. Triangles indicate the catalytic residues.
(TIF)

S1 Raw images (TIF)
