# Supplementary figures and images for: Novel silkworm (Bombyx mori) sulfotransferase swSULT ST3 is involved in metabolism of polyphenols from mulberry leaves
Source: PLoS One. 2022 Aug 4;17(8):e0270804. doi: 10.1371/journal.pone.0270804 (PMC9352109; doi:10.1371/journal.pone.0270804)

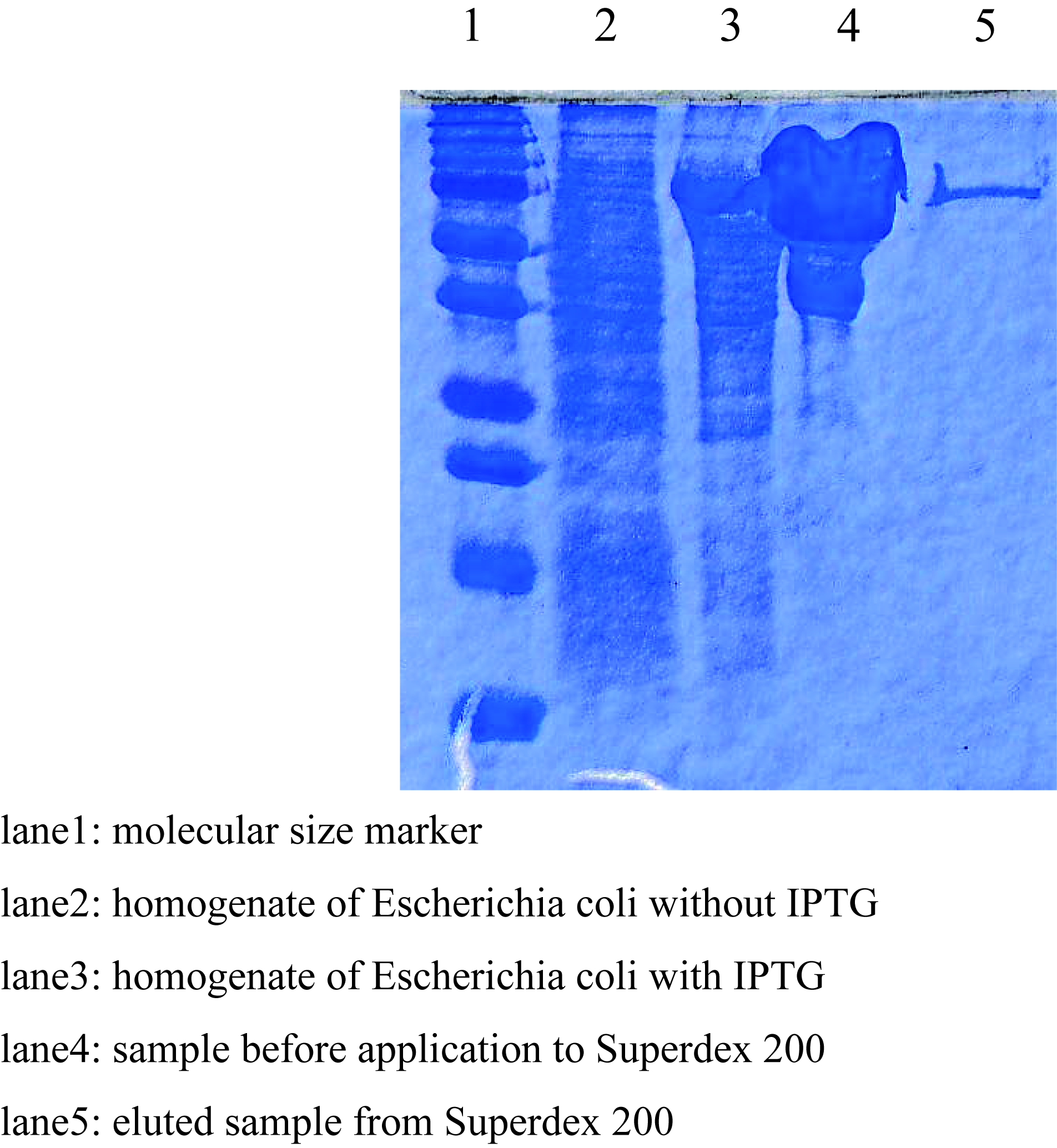

Supplement: S1 Raw images — (TIF) [file pone.0270804.s002.tif]
